# Mechanisms of action of antimicrobial peptides ToAP2 and NDBP-5.7 against *Candida albicans* planktonic and biofilm cells

**DOI:** 10.1038/s41598-020-67041-2

**Published:** 2020-06-25

**Authors:** Jhones do Nascimento Dias, Calliandra de Souza Silva, Alyne Rodrigues de Araújo, Jessica Maria Teles Souza, Paulo Henrique de Holanda Veloso Júnior, Wanessa Felix Cabral, Maria da Glória da Silva, Peter Eaton, José Roberto de Souza de Almeida Leite, André Moraes Nicola, Patrícia Albuquerque, Ildinete Silva-Pereira

**Affiliations:** 1 Department of Cell Biology, Institute of Biological Sciences, University of Brasília, Brasília, Brazil; 2 Biotechnology and Biodiversity Center Research, Biotec, Federal University of the Delta of Parnaíba, Parnaíba, Piauí Brazil; 3 Center for Research in Applied Morphology and Immunology, NuPMIA, Faculty of Medicine, University of Brasilia, Brasilia, Brazil; 4 Faculty of Medicine, University of Brasília, Brasília, Brazil; 5 Faculty of Ceilândia, University of Brasília, Brasília, Brazil; 6 LAQV/REQUIMTE, Department of Chemistry and Biochemistry, Faculty of Sciences of the University of Porto, Porto, Portugal

**Keywords:** Drug discovery, Microbiology

## Abstract

*Candida albicans* is a major cause of human infections, ranging from relatively simple to treat skin and mucosal diseases to systemic life-threatening invasive candidiasis. Fungal infections treatment faces three major challenges: the limited number of therapeutic options, the toxicity of the available drugs, and the rise of antifungal resistance. In this study, we demonstrate the antifungal activity and mechanism of action of peptides ToAP2 and NDBP-5.7 against planktonic cells and biofilms of *C. albicans*. Both peptides were active against *C. albicans* cells; however, ToAP2 was more active and produced more pronounced effects on fungal cells. Both peptides affected *C. albicans* membrane permeability and produced changes in fungal cell morphology, such as deformations in the cell wall and disruption of ultracellular organization. Both peptides showed synergism with amphotericin B, while ToAP2 also presents a synergic effect with fluconazole. Besides, ToAP2 (6.25 µM.) was able to inhibit filamentation after 24 h of treatment and was active against both the early phase and mature biofilms of *C. albicans*. Finally, ToAP2 was protective in a *Galleria mellonella* model of infection. Altogether these results point to the therapeutic potential of ToAP2 and other antimicrobial peptides in the development of new therapies for *C. albicans* infections.

## Introduction

*Candida albicans* is a fungal species present in the normal human microbiota, colonizing several areas of the body. However, under certain circumstances, this species may become a pathogen, causing diseases that can be life-threatening^1–4^. The use of broad-spectrum antibiotics, immune suppression, or changes in the local host environments are examples of situations that may favor the proliferation of *C. albicans* and the onset of disease^5–8^. Moreover, *C. albicans* ability to thrive in human tissues involves metabolic and morphological changes associated with the expression of different virulence factors^9^.

*C. albicans* virulence factors include secretion of enzymes, adhesion to cell surfaces and evasion of the immune system^10,11^. Two virulence factors of major clinical importance are the fungal polymorphism and its ability to form biofilms^12–14^. *C. albicans* ability to transit between yeast and filamentous forms is crucial for pathogenesis and both fungal forms are relevant for infection^15^. For instance, hyphae have a major role on tissue invasion, whereas the yeast morphology facilitates fungal dispersion^16^. The different fungal morphologies are also important for the formation of *C. albicans* biofilms^17^. Living in biofilms confers to the microorganisms several advantages, when compared to the planktonic lifestyle, including protection against immune cells, increased resistance to antimicrobials agents and other chemical, physical and environmental stressors^18,19^.

The number of antifungals currently available for clinicians is limited and the scenario is worsened by the rise of antifungal resistance to available drugs such as azoles, polyenes and echinocandins^20,21^. For example, *C. albicans* biofilms present resistance to fluconazole^6,22^, one of the most commonly used agents in the treatment of mucosal and superficial candidiasis^23^. In addition to resistance, many of the current systemic antifungal drugs are also toxic to host cells often producing important side effects. Altogether these factors stress the need of new therapeutic strategies against candidiasis and other mycoses^20^.

Antimicrobial peptides (AMPs) have been considered a promising alternative for the prevention and treatment of different infectious diseases^24–27^. AMPs are small, low-molecular-weight cationic peptides that are part of the innate immune response of the great majority of organisms^28–30^. In addition to their antimicrobial activity, natural and synthetic AMPs can also be immunomodulatory, modulating inflammation, chemotaxis and immune cell differentiation^31–33^. AMPs have been shown to be effective against bacteria, fungi, viruses and protozoa and are less prone to induce resistance because of their multiple cellular targets^34–37^.

Our group identified AMPs derived from a scorpion venom cDNA library presenting activities against different *Candida* spp and *C. neoformans*. Based on their potential antifungal activities, cationic antimicrobial peptides ToAP2, from a cDNA library of the scorpion *Tityus obscurus* venom gland (Uniprot entry LT576030); and NDBP-5.7, from a cDNA library of the scorpion *Opisthacanthus cayaporum* venom gland (Uniprot entry C5J886) were synthetized for further characterization in this work. ToAP2 (26 residues of amino acid, net charge +6) and NDBP-5.7 (13 residues of amino acid, net charge +1) presented MIC of 12.5 µM (37.5 µg/ml) and 25 µM (35.8 µg/ml) for *C. albicans* planktonic cells, respectively^38^. In addition, both are non-disulfide-bridged peptides (NDBP) belonging to NDBP subfamilies 3 and 5, respectively, according to the classification proposed by Zeng *et al*.^39^, who grouped scorpion NDBPs, in six subfamilies based on their pharmacological action, length and structural similarity. In this work we explored their mechanisms of action against *C. albicans* planktonic and biofilm cells and their activity in combination with two important antifungals, fluconazole and amphotericin B.

## Results

### Minimal inhibitory concentrations (MIC) for *C. albicans*

We have already evaluated the MIC of ToAP2 and NDBP-5.7 against *C. albicans* SC-5314 in our previously work using an inoculum of 2 × 10^3^ cells/mL. However, some assays described in this work, such as flow cytometry and Electron Transmission Microscopy (TEM), required a higher cell density or a non-filamenting strain. To solve the filamentation problem for the flow cytometry analysis, we used the non-filamenting strain SSY50-B^40^, which showed the same MIC values to both AMPs presented by the filamenting strain SC-5314 (12.5 µM for ToAP2 and 25 µM for NDBP-5.7)^38^. In addition to that, we evaluated ToAP2 and NDBP-5.7 MIC for both *C. albicans* strains at a cell density of 1 × 10^6^ cells/mL. The obtained MIC was also the same for both strains (25 µM for ToAP2 and 100 µM for NDBP-5.7).

### ToAP2 and NDBP-5.7 permeabilize *C. albicans* cell membrane

**Figure 1:**
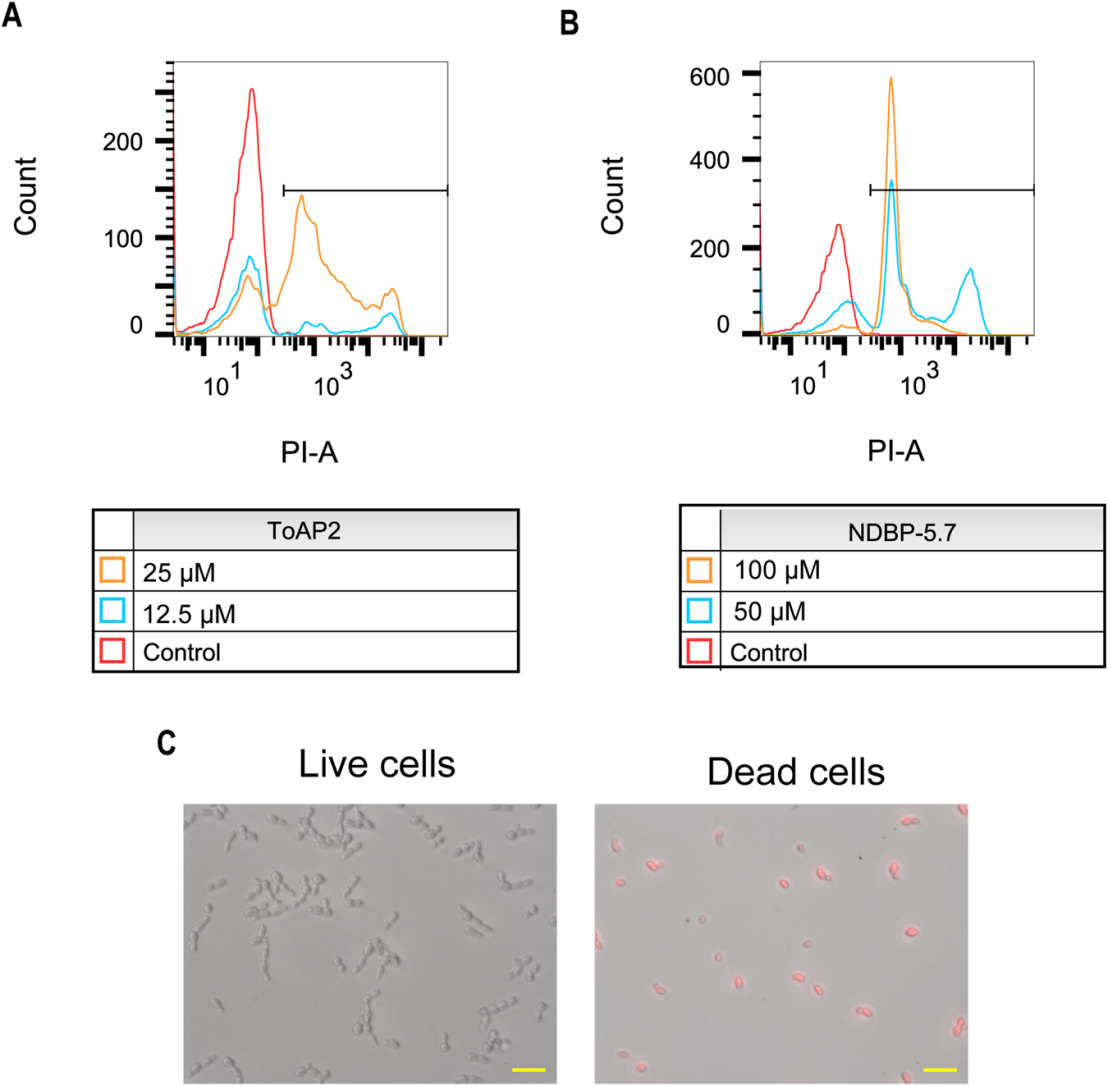
Flow cytometry analysis of *C. albicans* (SSY50-B strain) cell membrane integrity after treatment with ToAP2 and NDBP-5.7. After 2 h of treatment with ToAP2 (**A**) or NDBP-5.7 (**B**) at MIC and Sub-MIC concentrations the uptake of propidium iodide by fungal cells was measured by flow cytometry. Untreated cells were used as a control. The horizontal line indicates the PI-positive gate (**C**). Fluorescence microscopy of dead (ethanol permeabilized) and live cells (untreated) in the presence of PI. Scale bars 20 µm.

Plasma membrane integrity of *C. albicans* non-filamenting strain SSY50-B^40^ treated with both peptides was evaluated by flow cytometry using PI (Fig. 1). Both peptides led to a dose-dependent increase in the percentage of permeabilized cells. Concentrations of ToAP2 from 12.5 μM (subMIC) to 25 μM (MIC) produced an increase of respectively 42.1% to 65.8% in the proportion of permeabilized cells in comparison to the control (Fig. 1A). While the treatment with 50 μM (subMIC) and 100 µM (MIC) of NDBP-5.7 increased the percentage of permeabilized cells in 66,87% and 88,97%, respectively (Fig. 1B). Figure 1C compares live and dead cells in the presence of PI showing that only the dead cells emit the fluorescent signal.

### ToAP2 alters the ultrastructure of *C. albicans*

**Figure 2:**
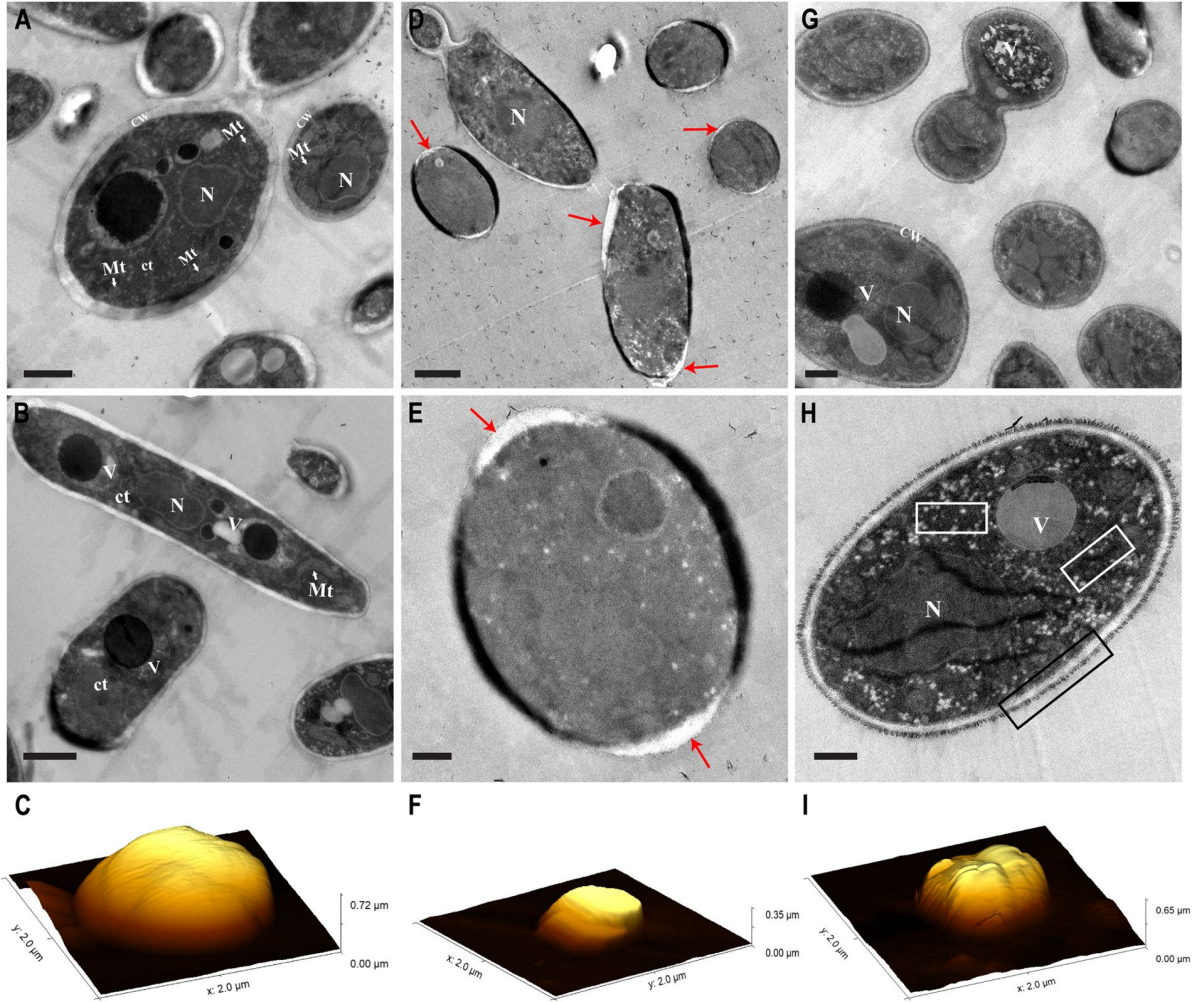
TEM and AFM images of *C. albicans* untreated and treated cells with ToAP2 and NDBP-5.7 at sub-inhibitory concentrations. (**A–C**) control cells; (**D–F**) and (**G–I**) ToAP2 and NDBP-5.7 treated cells, respectively. TEM images are indicated by the letters (**A,B,D,E,G,H**) and AFM by the letters (**C,F,I**). Scale bars = 1 µm (**A,B,D,G**) and 0.5 µm (**E,H**). Red arrows indicate regions of discontinuity of the cell wall/cell membrane. White square regions indicate accumulation of tiny vacuoles. Black square region – indicates the fibrillar material at the cell wall of NDBP-5.7-treated cells. CW – Cell wall, Mt – Mitochondria, N – Nucleus, Ct – Cytoplasm, V -Vacuole.

The ultrastructure of *C. albicans* cells treated with AMPs ToaP2 and NDBP-5.7 was evaluated by transmission electron microscopy. Control samples revealed cells with preserved morphology and cell shape surrounded by a regular cell wall and continuous cell membrane. These cells also presented a homogeneous cytoplasm with nucleus, mitochondria and other membrane-enclosed organelles (Fig. 2A,B). On the other hand, ToAP2 treated cells show several ultrastructure alterations, including an irregular thickness and shape of the cell wall, regions of discontinuity of the cell wall and cell membrane and an amorphous and diffuse cytoplasm lacking a clear organization of organelles (Fig. 2D,E). This could suggest that in addition to the cell membrane, this peptide could be also acting on intracellular membranes. In contrast, cells treated with NDBP-5.7 displayed a more regular morphology, especially at the cell wall organization (Fig. 2G,H), although we could also observe some cells showing an irregular cytoplasm filled with a large number of tiny vacuoles (Fig. 2H). Interestingly, we could also observe a very distinguishable cell wall fibrillar outer layer in NDBP-5.7 treated cells that was not clear in the control cells.

### ToAP2 and NDBP-5.7 induce morphological changes in *C. albicans* cells at subinhibitory concentrations

We used atomic force microscopy to visualize possible alterations in *C. albicans* cell surface or morphology induced by a 24 h treatment with both peptides. Control cells not exposed to any of the AMPs showed the typical oval and well-defined morphology and a smooth surface. Treatment with sub-inhibitory concentration of ToAP2 (6.25 μM) and NDBP-5.7 (25 μM) resulted in *C. albicans* cells with altered cell morphologies in comparison to control cells (Fig. 2C). In overall, the most noticeable changes in ToAP2-treated were deformations in their area including flattened regions in their surface instead of the round morphology observed in the control cells. On the other hand, NDBP-5.7-treated cells had a wrinkled appearance with significant increase in cell-surface deformations (Fig. 2F,I). Results from both microscopy approaches combined with the results of the membrane permeability assay suggest that the cell surface is probably a target for both AMPs.

### ToAP2 inhibits *C. albicans* filamentation

**Figure 3:**
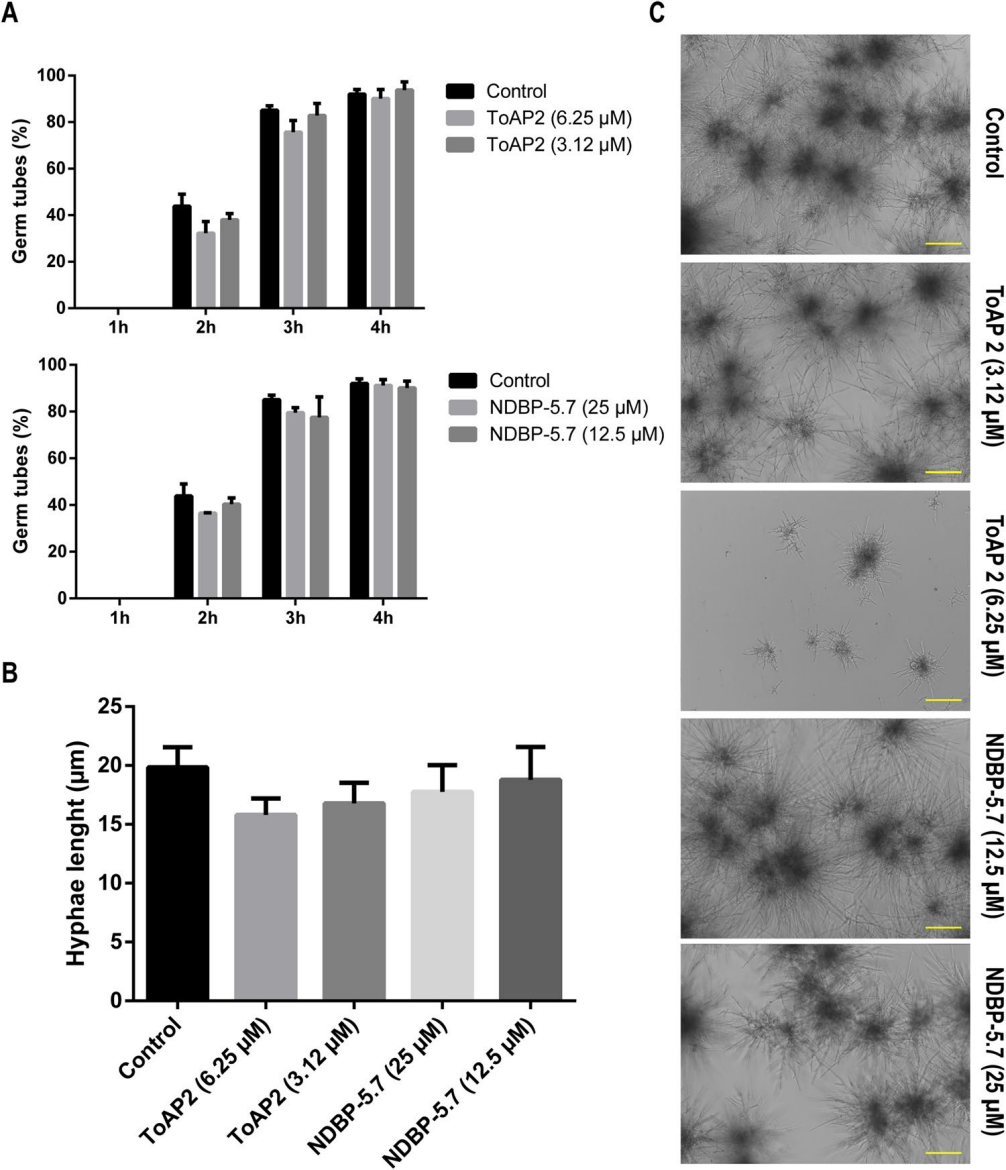
Effects of ToAP2 and NDBP-5.7 on *C. albicans* filamentation. (**A**) Percentage of filamenting cells during 4 h of treatment with different concentrations of ToAP2 or NDBP-5.7. (**B**) Length of *C. albicans* filamenting cells after 3 h of treatment with ToAP2 or NDBP-5.7. (**C**) Bright field microscopy of the effects of ToAP2 and NDBP-5.7 on *C. albicans* filamentation after 24 h treatment. Mean ± SEM (p ≤ 0.05). Scale bars 100 µm.

Sub-inhibitory concentrations of ToAP2 (6.25 and 3.12 μM) and NDBP-5.7 (12.5 and 25 μM) were used to evaluate their effect on *C. albicans* SC 5314 filamentation during 4 h or 24 h treatments. There was no difference in the percentage of fungal cells with filaments or in the germ-tube lengths in comparison to the control at the early time points (Fig. 3A,B). In contrast, there was a decrease in fungal filamentation after 24 h of treatment with ToAP2 (Fig. 3C). Considering that filamenting cells normally grow in clumps, impairing the evaluation of the size of individual cells or the number of individuals cells in a clump at later time points, we complement the microscopic analysis presented in Fig. 3C by time-lapse microscopy of cells submitted to the different treatments along 24 h (Supplementary Movie S1). At MIC concentrations we observed very little cell filamentation in response to both peptides, probably because the cells are dying. Interestingly, the treatment with 6.25 μM of ToAP2 not only delayed *C. albicans* filamentation, but also decreased de overall length of the filaments in comparison to the control, apparently without any major effect on cell viability (Supplementary Movie S1). Although NDBP-5.7 apparently have not affected the filamentation on the tested conditions, at the second half of the Supplementary Movie S1, we can still see several yeast cells lacking germ-tubes.

### Combined action of antifungal drugs with ToAP2 and NDBP-5.7

The combined activity of antifungal agents and AMPs was evaluated using a Checkerboard assay^41^, based on the MIC concentrations of each AMP^38^.

The results revealed that ToAP2 (3.12 µM for the assays with fluconazole or 0.78 µM for the assay with amphotericin B) presented a synergistic effect when combined with fluconazole (0.25 µM) and amphotericin B (0.13 µM), with FIC indices of 0.5 and 0.182, respectively. NDBP-5.7 (1.56 µM) was also synergic with amphotericin B (0.13 µM) showing a ΣFIC of 0.182), but only additive with fluconazole (ΣFIC of 0.562). In the association between the peptides, the best combination of ToAP2 (6.25 µM) with NDBP-5.7 (6.25 µM) showed only additive effect (Table 1).

**Table 1:** Combined activity of AMPs and antifungals.

Drug A	Drug B	FICa/µM	FICb/µM	∑FIC	Action
Fluconazole	ToAP2	0.25/0.20	0.25/3.12	0.5	Synergism
	NDBP-5.7	0.5/0.40	0.062/1.56	0.562	Additive
Amphotericin B	ToAP2	0.12/0.13	0.062/0.78	0.182	Synergism
	NDBP-5,7	0.12/0.13	0.062/1.56	0.182	Synergism
ToAP2	NDBP-5,7	0.5/6.25	0.25/6.25	0.75	Additive

FIC values: ≤ 0.5= Synergism; 0.5 < FIC ≤ 1.0 = Additive; 1.0 < FIC ≤ 2.0 = indifferent; >2 = antagonism.

### ToAP2 is effective in different stages of biofilm formation

**Figure 4:**
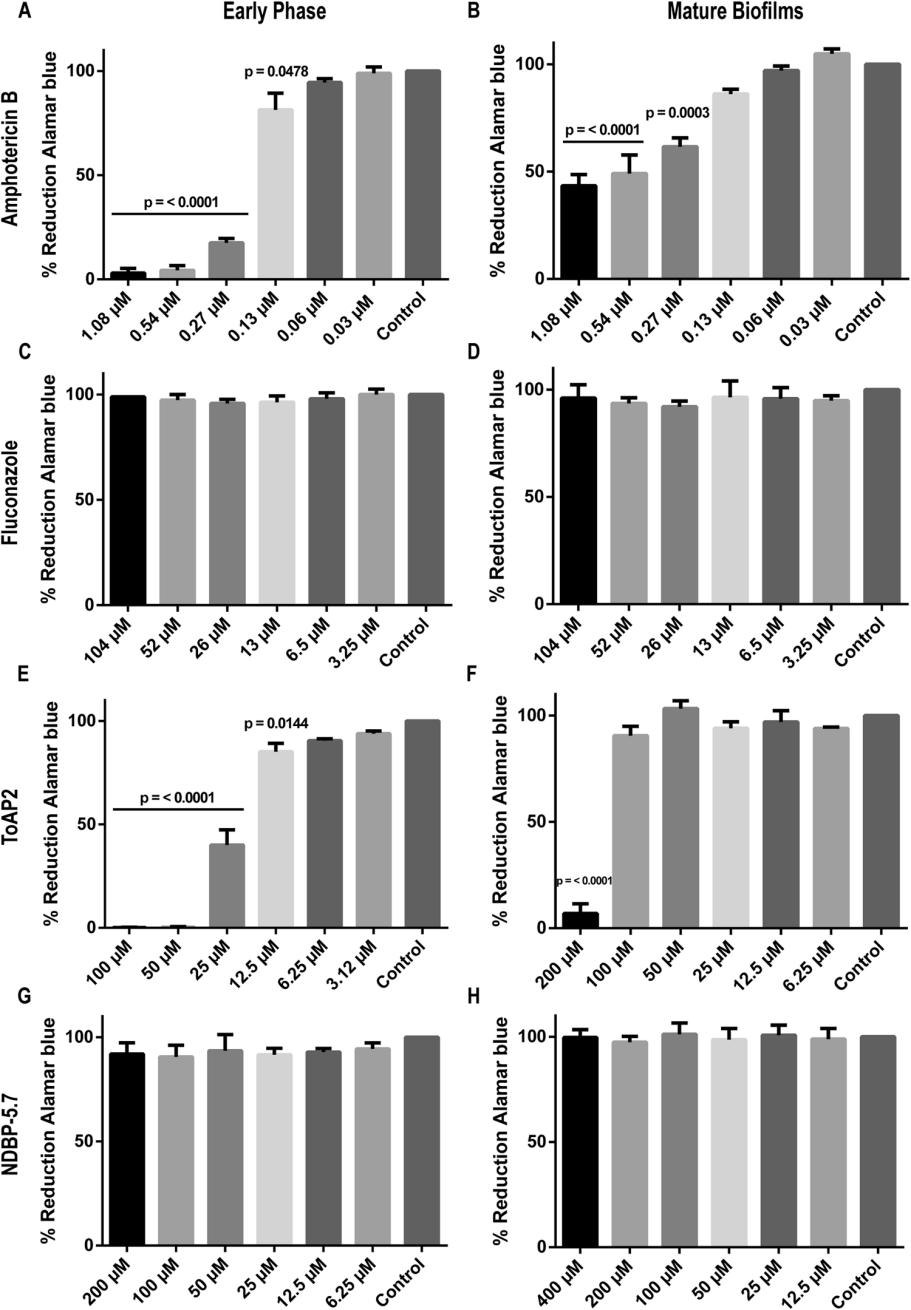
Effects of ToAP2 and NDBP-5.7 on the viability of early phase (4 h) or mature (24 h) *C. albicans* biofilms. *C. albicans* biofilms previously grown for 4 h (**A,C,E,G**) or 24 h (**B,D,F,H**) were treated for 24 h with different concentrations of amphotericin B (**A,B**), fluconazole (**C,D**), ToAP2 (**E,F**) or NDBP-5.7 (**G,H**). After that, cell viability was evaluated using the alamarBlue reduction assay. Results were expressed as a mean of three independent assays. P-value indicates statistical difference using the Tukey *post-test*. The results represent mean ± SEM.

We evaluated the effects of ToAP2 and NDBP-5.7 at early-phase (after 4 h of attachment) and mature biofilms (after 24 h of attachment). Treatment with amphotericin B concentrations equal or higher than 0.27 μM produced an inhibition of at least 75% in the viability of early-phase biofilms. Although the effects were less pronounced, the three highest concentrations of amphotericin also produced a 40–60% inhibition of mature biofilms. (Fig. 4A,B). In contrast, fluconazole had no activity at any of the tested concentrations at both phases of biofilm formation (Fig. 4C,D).

ToAP2 displayed significant antimicrobial activity against both early-phase (p = <0.0001) and mature biofilm phases (p = <0.0001) (Fig. 4E,F). Starting with 25 μM of ToAP2 we observed a dose dependent inhibition of early phase biofilms ranging from 60.02 to 99.85%. ToAP2 also produced a 97.02% decrease in the viability of mature biofilms, but only at 200 μM. On the other hand, NDBP-5.7 peptide showed no activity in either phases of *C. albicans* biofilms (Fig. 4G,H). The viability of biofilms after any of the treatments was also evaluated by fluorescence microscopy using the viability dye Phloxine B (Supplementary Figs. S2 and S3).

### Peptides ToAP2 and NDBP-5.7 permeabilize the membrane of biofilm cells

**Figure 5:**
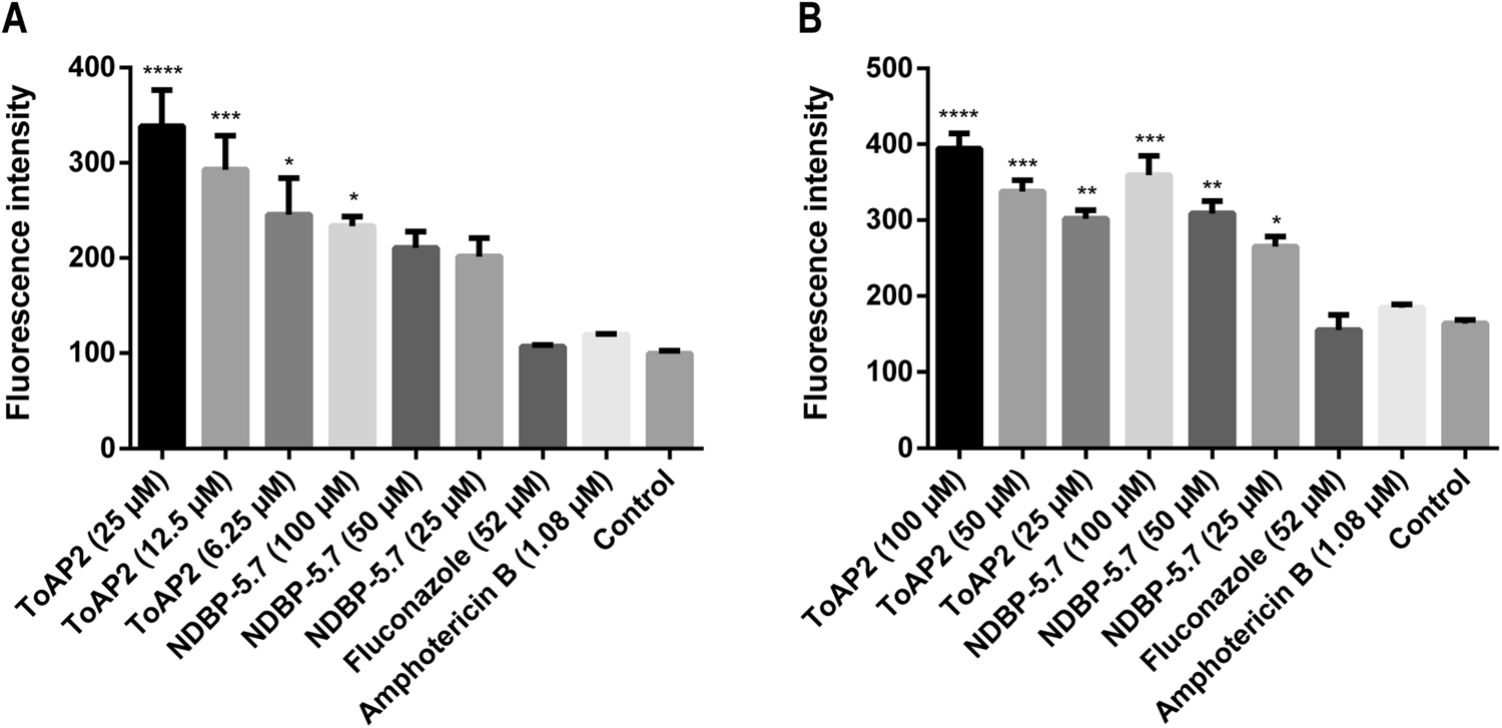
Effects of AMPs on the membrane integrity of *C. albicans* biofilm cells. Alterations in membrane permeability was evaluated using an adaptation of the Rhodamine 6G efflux assay after 24 hours of treatment with different concentrations of ToAP2 and NDBP-5.7 in early phase (**A**) or mature biofilm (**B**). Untreated cells and cells treated with fluconazole or amphotericin B were used as a control. P-value indicates statistical difference by Tukey post-test (****p ≤ 0.0001; ***p ≤ 0.001; **p ≤ 0.01; *p ≤ 0.05). Mean ± SEM.

Membrane integrity of *C. albicans* biofilm cells treated with ToAP2 or NDBP-5.7 was evaluated using an adaptation of the fluorophore Rhodamine 6G efflux assay as described before^42^. Both peptides produced a dose-dependent increase in permeabilization of *C. albicans* biofilm cells at both phases of biofilm formation. There was an increase in the permeabilization of *C. albicans* early-phase biofilm cells with concentrations of 25–6.25 µM of ToAP2 and of 100–25 µM of NDBP-5.7 (Fig. 5A; p = 0.0002). Similarly, we observed an increase in the permeabilization of mature biofilm cells after treatments with concentrations of 100–25 µM of either ToAP2 or NDBP-5.7 (Fig. 5B; p = <0.0001). Neither amphotericin B (1.08 µM) or fluconazole (52 µM) displayed significant differences in the permeabilization of biofilm cells compared to the untreated control at the tested concentrations.

### Effect of ToAP2 on the survival of *Galleria mellonella* infected with *C. albicans*

**Figure 6:**
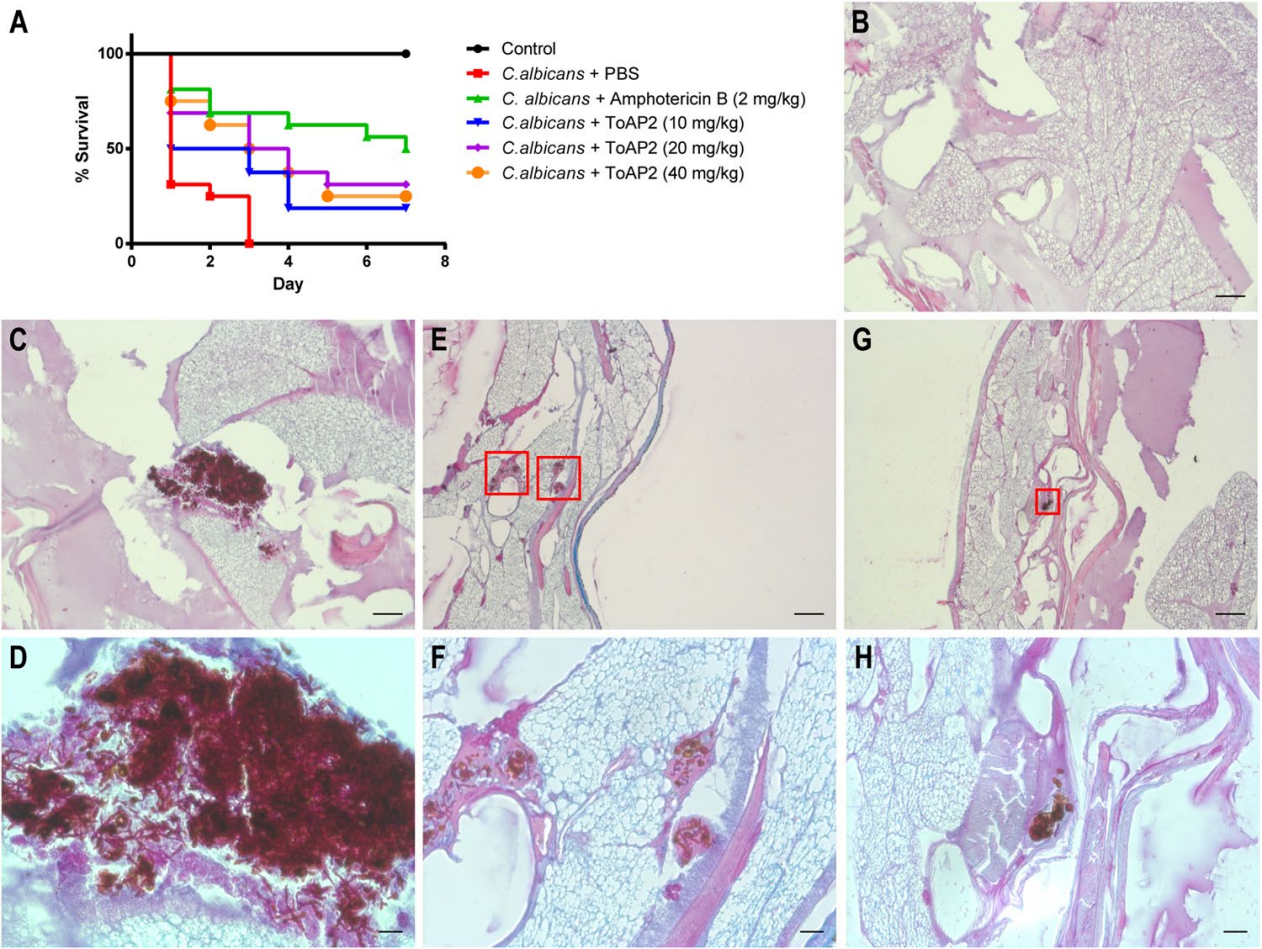
ToAP2 Effects on *G. mellonella* infected with *C. albicans*. (**A**) Survival curve of *G. mellonella* infected with *C. albicans* and treated with amphotericin B or different concentrations of ToAP2 (**A**). Histopathology of *C. albicans* infection in the *G mellonella* model. After 1 h of infection, the larvae were treated with doses of ToAP2 at 40 mg/kg (**E,F**) and Amphotericin at 2 mg/Kg (**G,H**). Uninfected (**B**) and untreated (**C,D**) larvae were used as controls. Panel H is a higher magnification of the region depicted on panel G. Black arrows indicate the presence of foci of *C. albicans*. Figure B, C, E and G scale bar 100 µm; Figure D, F and H scale bar 20 µm. Graphics were generated from two independent assays using Kaplan-Meier survival curves. The squares in panels E and F indicate fungal cells.

The ToAP2 toxicity and antimicrobial activity *in vivo* was evaluated using the *G. mellonella* model. For the toxicity evaluation different groups of larvae were injected with 10, 20 and 40 mg ToAP2/kg or with 2 mg/kg of Amphotericin B. None of the AMP doses were toxic to *G. mellonella* (data not shown). *In vivo* antimicrobial activity of ToAP2 was evaluated after infecting different groups of larvae with *C. albicans* and treating the groups with different doses of this peptide. All doses of ToAP2 resulted in an increased survival of the larvae when compared to control group (treated with PBS) (Fig. 6A). Doses of 10, 20 and 40 mg/kg resulted in an increased survival rate of 18.7, 31.2 and 25% respectively. ToAP2 doses of 20 and 40 mg/kg resulted in survival rates not statistically different when compared to 2 mg/kg of Amphotericin B, which increased larvae survival to 50%. In the larvae group treated with PBS (control group), all organisms died in the third day after *C. albicans* infection.

### ToAP2 treatment decreases fungal burden of *G. mellonella* larvae infected with *C. albicans*

To evaluate the *in vivo* effects of ToAP2 we performed histopathology analysis of *G. mellonella* larvae infected with *C. albicans* and treated with this peptide, amphotericin B or PBS (untreated control). Non-infected larvae were used as control (Fig. 6A). Infected and untreated larvae (Fig. 6B) revealed dissemination of *C. albicans* yeast and hyphae cells throughout various parts of the organism (Fig. 6C,D). Larvae treated with either 40 mg/kg ToAP2 (Fig. 6E,F) or 2 mg/kg of Amphotericin (Fig. 6G,H) showed fewer foci of infection with lower number of fungal cells and most of them in the yeast morphology, although the effects were more accentuated after amphotericin B treatment.

## Discussion

The structural and physiological similarity between fungal and mammalian cells makes the development of new antifungals a complex challenge^43^. Given this, the AMPs are small conserved molecules part of the innate immune response of several organisms and a promising group of new antimicrobial agents^44^. In the present work, we further evaluated the antifungal activity of two AMPs previously described and partially characterized by our group, ToAP2 and NDBP-5.7. We analyzed the activity of both AMPs on membrane integrity, cell morphology, yeast-to-hypha transition, and biofilm formation of *C. albicans*.

Flow cytometry analysis of propidium iodine labeled cells revealed that treatment with both peptides produced a dose-dependent increase in membrane permeability and cell death. A similar permeabilization of *C. albicans* cells was previously described after treatment with Cathelicidin PMAP-36 analogues^45^. AFM and TEM results also suggested the induction of membrane damage and loss of cell surface integrity by both peptides, particularly by ToAP2. AFM images of fungal cells treated with ToAP2 and NDBP-5.7 showed morphological alterations including the loss of the typical oval shape and an increase in surface roughness, when compared to untreated control cells. Those cell deformations might be linked to leakage of cellular contents as a result of cell membrane damage. Comparable effects were observed in other AFM studies describing alterations in *C. albicans* cell morphology and membrane integrity when in the presence of antimicrobial peptides Psd1^46^ and LL-37^47^. TEM of *C. albicans* treated with ToAP2 revealed several cells showing a loss of cell wall integrity and presenting an internal amorphous cytoplasm with mostly no distinguishable organelles, suggesting that this AMP can induce damage not only on the plasma membrane but also in the membrane of cellular organelles inducing loss of integrity of those structures. These alterations bear similarities to the ones produced by the treatment of *C. albicans* cells with the phosphorylated peptide K40H derived from the constant region of human IgMs^48^ or with the antifungal peptide AMP MCh-AMP1 derived from chamomille (*Matricaria chamomilla*)^49^.

These observations suggest that membrane damage and permeabilization is one of the mechanisms of action of those molecules as previously reported for many antimicrobial peptides^50,51^. Our findings are also in agreement with previously published circular dichroism analyses showing ToAP2 and NDBP-5.7 tendency to form α-helix structures^38^, a common feature in other antimicrobial peptides targeting microbial cell membranes^52–54^. However, we cannot exclude other mechanisms of action for these molecules, as AMPs have been shown to have multiple antifungal actions such as DNA damage, inhibition of protein synthesis or metabolic pathways and induction of apoptosis or oxidative stress in addition to membrane permeabilization^50,51^.

An important factor associated to *C. albicans* pathogenesis is its ability to perform yeast-to-hypha transition^8^. An alternative therapeutic approach for infectious diseases is targeting microbial virulence attributes, what would increase microbial susceptibility to antimicrobials or to the immune system components with lower chances of inducing microbial resistance^55^. As a result, many research groups have dedicated efforts in the search of molecules that could inhibit *C. albicans* filamentation^56–58^.

As shown in Fig. 3, subinhibitory concentrations of both AMPs presented no effect on the percentage of *C. albicans* cells presenting germ tubes formation during the first four hours of treatment. However, a prolonged incubation with 6.25 µM of ToAP2 significantly delayed filamentation and reduced the number of cells presenting germ tubes without killing the cells (Fig. 3C). It is important to notice that filamentation is a complex process dependent of many different factors, such as availability of nutrients and the presence of serum in the medium, and all of them can interfere with the peptide inhibitory effects^59^.

ToAP2 showed the most promising antimicrobial effects in most of our assays. However, as other AMPs^60,61^, this peptide presents some toxicity to mammalian cells in concentrations close to its MIC for *C. albicans* and other tested fungi^38^. One approach to overcome this problem is the optimization of this peptide sequence aiming to decrease its toxicity to mammalian cells/enhance its antimicrobial activity. Another approach could be the combined use of AMPs with conventional antifungals. The use of drug combinations is a strategy to improve drug’s efficacy reducing their concentrations to non-toxic levels and at the same time a strategy to decrease resistance development^62^.

Thus, we evaluated the combined actions of ToAP2 and NDBP-5.7 with each other and with amphotericin B or fluconazole against planktonic cells of *C. albicans*. The combination of ToAP2 and NDBP-5.7 AMPs showed an additive effect, while both peptides presented a synergistic effect with amphotericin B and ToAP2 also with fluconazole (Table 1). In all tested combinations, the active concentrations used were reduced in comparison with the MIC of each of the compounds alone^38^. For instance, the combination of AMPs with amphotericin B produced a reduction of up to 4X in their MIC. Our results were similar to those found by Lum *et al*.^63^, who found that the combination of antimicrobial peptides with amphotericin B resulted in a synergistic effect, while the combination between peptides was additive or indifferent in *C. albicans* infection models. The peptides MUC7 12-mer, hepcidin 20 and defensin HsAFP1 have also shown synergism with antifungal drugs^64–67^. Our results might also suggest that ToAP2 and NDBP-5.7 act on different targets than the antifungal agents, thus their complementary effect as previously suggested for other AMPs^68^. According to Gray and colleagues^69^, the main mechanism of action of Amphotericin B in yeast cells (*Saccharomyces cerevisiae* and *C. albicans*) is the binding and sequestration of ergosterol, while membrane permeabilization is a secondary mechanism not necessarily required for its fungicidal effects. In addition to its activities at the membrane level, another important microbicidal activity of amphotericin B is the induction of reactive oxygen species (ROS) accumulation observed in many fungal species. It is believed that this mechanism could be related to the low induction of fungal resistance to this compound^70^. On the other hand, fluconazole, a fungistatic azole, interacts with a key enzyme in fungal sterol biosynthesis inhibiting the synthesis of ergosterol and compromising membrane permeability and fluidity^71^. Microbial cell membrane is also regarded as the primary target of AMPs, being membrane permeabilization probably by pore formation one of the main effects of those molecules. Our flow cytometry and microscopy results point to the same happening in *C. albicans* cells treated with NDBP-5.7 and ToAP2. However, as we mentioned before, we cannot exclude other mechanisms of actions for those molecules and further studies are needed to elucidate the mechanisms behind the synergistic effects we have observed.

Compared to planktonic cells, biofilms can be up to 1000 times more resistant to antimicrobials, due to certain factors such as the presence of a dense extracellular matrix, changes in cell metabolism and the positive regulation of efflux pumps among others^72–74^. Consequently, few antimicrobials capable of treating biofilm-related infections are available today^75^. Our results indicate that ToAP2 presented inhibitory action on both phases of biofilm formation. Amphotericin B but not fluconazole was also active in our tests, as previously reported^63,76–79^.

Biofilm resistance to fluconazole could be partially explained by the presence of the extracellular matrix, composed by proteins, polysaccharides, nucleic acids and lipids, which serves as a barrier for antifungals^80,81^. It has been reported that β-1,3 glucan, present in *C. albicans* biofilm matrix, can hijack triazoles preventing these drugs from reaching cell membrane^82,83^. Both ToAP2 and NDBP-5.7 were able to permeabilize *C. albicans* biofilm cell membrane in both phases of biofilm formation (Fig. 5A,B). Interestingly, NDBP-5.7 affected membrane permeabilization without decreasing the viability of the biofilm (Fig. 4G,H).

We have shown that ToAP2 was not lethal to *G. mellonella* larvae when used at concentrations up to 40 mg/kg, similar to what was observed with AMPs hMUC7–12, hLF(1–11), DsS3(1–16) by Maccalum *et al*.^64^. ToAP2 doses of 10, 20 and 40 mg/kg were also able to significantly increase larva survival after *C. albicans* infections in our assays. At the two highest concentrations of this AMP the larva survival profile was similar to what we observed with the amphotericin B treatment. Although amphotericin B effects were more pronounced, histopathological analysis of larvae infected with *C. albicans* and treated with any of the two compounds resulted in a reduction in the number of foci of infection and a more limited infection in contrast with the disseminated form observed in the control (Fig. 6). In addition, in the control groups, we observed the presence not only of yeast cells as in the treated-groups, but also filament cells possibly indicating *C. albicans* tissue invasion as described before^84^. These results are also in accordance to previously described works showing the efficacy of antifungals in the treatment of *G. mellonella* infected with *C. albicans*^85–87^. Finally, the results observed with ToAP2 indicated a possible systemic action of this peptide in this *in vivo* infection model.

In conclusion, we have shown that the peptides ToAP2 and NDBP-5.7 are active against planktonic and biofilms of *C. albicans*. Treatment with these peptides increased cell permeability and generated important morphological alterations in *C. albicans* cells. ToAP2 showed the most promising antimicrobial effects in most of our assays an can be considered a putative candidate for new antifungal formulations, especially for topical treatments or to be used in combined therapeutic strategies. The observed synergy of this peptide with fluconazole and amphotericin B is particularly encouraging because we could observe an increase in the efficacy of both molecules decreasing their active concentrations and probably also their toxicity to mammalian cells.

Invasive candidiasis affects around 750 thousand people yearly. This disease has a close association with health-care environment where is a major cause of morbidity and mortality specially among elderly and premature babies^88,89^. As for other invasive fungal infections the available therapeutic options are extremely limited and associated with high toxicity, costs and the increase in fungal strains resistant to the few antifungal drugs currently available, such echinocandins and azoles^90–93^.

The results described in this work are promising in view of the urgent need for alternative antifungal molecules to treat systemic mycoses and to prevent the development of fungal resistance. Currently, we are working on the design and optimization of the antimicrobial features of ToAP2, aiming to circumvent its cytotoxicity against mammalian cells and to increase the biological properties reported here.

## Material and methods

### Synthesis of antimicrobial peptides

ToAP2 (FFGTLFKLGSKLIPGVMKLFSKKKER) and NDBP-5.7 (ILSAIWSGIKSLF-NH2) sequences are derived from cDNA libraries made from the venom glands of *Tityus obscurus* and *Opisthacanthus cayaporum* scorpions^38^. The AMPs were chemically synthesized by Biomatik using an Fmoc / t-butyl on solid support strategy. Peptide purification, characterization and purity evaluation were performed as previously described by Guilhelmelli^38^.

### Fungal culture conditions

*C. albicans* strain SC 5314 was used in most assays, except in the experiments of flow cytometry and Atomic Force Microscopy. In these assays we used the non-filamenting strain *C. albicans* SSY50-B, kindly provided by Stephen P. Saville and José L. Lopez-Ribot^40^. Fungal cells from frozen stocks were grown in Sabouraud dextrose broth for 18 h at 30 °C and 200 rpm. After that, cells were harvested by centrifugation (1000 g at 25 °C), washed three times in sterile phosphate buffer (PBS), counted in a hemocytometer and diluted to the appropriate cell densities.

### Minimum inhibitory concentration (MIC) evaluation

As previously described by our group^38^, all antifungal assays were carried out according to guidelines of the broth microdilution susceptibility test by the Clinical and Laboratory Standards Institute (CLSI) M27-A3 with some modifications^94^. Briefly, cells suspensions of *C. albicans* cells at 2 × 10^3^ cells/mL were tested using concentrations of 100 to 0.78 µM of each AMP. The criterium to define the minimum inhibitory concentration (MIC) was the lowest AMP concentration that completely inhibited visible fungal growth at the end of the incubation period. All assays were done in biological triplicates performed on separate dates.

Before performing assays requiring a higher number of cells, such as the evaluation of cell membrane integrity or microscopic analyses, we determined the MIC for *C. albicans* suspensions at the cell density used in those experiments - 1 × 10^6^ cells/mL. Therefore, the higher MIC values for all antifungals in those assays.

### Evaluation of cell membrane integrity

An inoculum of 500 µl of 1 × 10^6^ cells/mL of *C. albicans* SSY50-B strain was added to RPMI medium in the presence or absence of different concentrations of ToAP2 or NDBP 5.7 and incubated in a shaker at 37 °C and 200 rpm for 90 minutes^45^. Afterwards, the cultures were incubated with 1 μg/mL propidium iodide (PI) for an additional 30 minutes, in the dark, at 37 °C. The cells were then analyzed on a BD FACSVerse flow cytometer (minimum of 20,000 events per sample). The resulting data was analyzed using the *FlowJo X* software. The gating strategy consisted of: a) gating on fungal cells on a forward scatter (area) x side scatter (area) plot and b) doublet exclusion by gating on a forward scatter (width) x forward scatter (height) plot. Live untreated cells, and 70% ethanol-treated (dead) cells were also incubated with PI and used as controls. The cells were observed in an inverted microscope (Axio Observer Z1 Carl Zeiss Microscopy, USA) (excitation: 546/12 nm; emission: 607/80 nm).

### Evaluation of ultrastructure by Transmission electronic microscopy

An inoculum of 1 × 10^6^ cells of *C. albicans* SSY50-B strain was added to RPMI with concentrations of 25 and 50 µM of ToAP2 and NDBP-5.7 respectively following standard protocol with modifications^45^. After 24 h, the cells were washed with PBS and cell suspensions were fixed by immersion in 2.5% glutaraldehyde and 2% paraformaldehyde in 0.1 M sodium cacodylate buffer (pH 7.4) solution for 2 days. After washing, the cells were post-fixating with 2% osmium tetroxide in 0.1 M sodium cacodylate buffer and incubated overnight with 1% uranyl acetate after a new wash. Then the cells were dehydrated in graded series of ethanol and soaked in Epon (EMS). The blocks were cut at 50 nm on a RMC Ultramicrotome (PowerTome, USA) and recovered into 200 mesh copper grids, followed by double contrast method with 2% uranyl acetate. Visualization was performed at 80 kV in a JEOL JEM 1400 microscope (Japan) and digital images were acquired using a CCD digital camera Orious 1100 W (Tokyo, Japan).

### Morphological analysis by atomic force microscopy (AFM)

Morphological evaluation was performed as described by Rodrigues de Araujo^95^ with modifications. An inoculum of 100 µL of 1 × 10^4^ cells/mL of *C. albicans* SSY50-B strain was incubated at 37 °C for 24 h with AMPs ToAP2 (6.25 µM) and NDBP-5.7 (25 µM) as described previously. Then, 20 μL of each culture was deposited in a glass slide and incubated at 35 ± 2 °C for 20 min. The effect of the peptides on fungal cells was evaluated on a TT-AFM microscope from AFM Workshop (USA) in tapping mode (vibrating) using a silicon cantilever (AppNano, USA) with a resonance frequency of approximately 353 kHz. The images (6 µm x 6 µm, 512 lines) were analyzed using the *Gwyddion 2.40 software*.

### Effect of AMPs on the *C. albicans* dimorphic transition

Filamentation assays using a suspension of *C. albicans* SC 5314 cells, in a final volume of 100 µL, were performed according to Yang^22^ and Derengowski^96^ with modifications. Briefly, in a 96-well polystyrene microplate, 1 × 10^4^ cells/mL fungal cells were inoculated in RPMI 1640 medium supplemented with L-glutamine and buffered to pH 7.0 with 165 mM MOPS, in the presence or absence of the peptides and cultivated at 37 °C. The cultures were photographed at different time points using an inverted microscope (Axio Observer Z1 Carl Zeiss Microscopy, USA). We took several pictures from each well of each experimental condition (at least 8) and a representative picture from each treatment was used for the figure. The number of cells showing germ-tubes and their average size were evaluated using the *ImageJ 1.51 s* software. The assay was performed in biological triplicates. Time-lapse microscopy was performed in the same microscope, with bright field images taken every 5 minutes for 24 h at 37 °C in the presence of at MIC and subMIC concentrations of ToAP2 (12.5 and 6.25 µM) and NDBP-5.7 (50 and 25 µM). The video was rendered in *Blender 3D* software at 15 fps.

### Effect of the combination of AMPs and conventional antifungal on planktonic *C. albicans*

Both peptides, amphotericin B (Stock solution 250 μg/mL in deionized water from Sigma-Aldrich USA) and fluconazole (Sigma-Aldrich USA) stock solutions were diluted in sterile MilliQ water. For the assays the molecules were used in the following concentrations: ToAP2 (12.5–0.39 µM); NDBP-5.7 (25–0.78 µM); amphotericin B (1.08–0.03 µM); fluconazole (1.62–0.05 µM).

The combinatory effects between both AMPs and also between each of them and fluconazole or amphotericin B were tested using the checkerboard assay with some modifications^41^. Cells of *C. albicans* SC 5314 (2 × 10^3^ cells/mL in 100 µL) were inoculated in RPMI 1640 in the presence of different concentrations of combined AMPs or AMPs plus antifungals. The plates were incubated at 37 °C for 24 h. After that, cell viability was evaluated using alamarBlue (Thermo Fisher Scientific) at 9% in a fluorescence plate reader with excitation at 550 nm and emission at 585 nm (SpectraMax M plate reader). This assay was performed in biological triplicates for each of the combinations tested. The synergistic action was evaluated by the Fractional Inhibitory Concentration (FIC).

$$\begin{array}{rcl}{\rm{FICa}} & = & \frac{{\rm{MICa}}\,{\rm{combined}}}{{\rm{MICa}}\,{\rm{isolated}}}\,{\rm{FICb}}=\frac{{\rm{MICb}}\,{\rm{combined}}}{{\rm{MICb}}\,{\rm{isolated}}}\\ \sum FIC & = & FICa+FICb\end{array}$$

We interpreted the FIC values obtained with the different drug combinations as synergistic (FIC ≤ 0.5), additive (0.5 < FIC ≤ 1.0), indifferent (1.0 < FIC ≤ 2.0) and antagonistic (FIC > 2.0)^97^.

### Antifungal action of AMPs against *C. albicans* biofilms

The antifungal activity of the AMPs against *C. albicans* SC 5314 biofilms was assessed using a microplate alamarBlue susceptibility assay as previously described by Repp *et al*.^98^. Briefly, a 100 µL inoculum containing 1 × 10^6^ cells/mL of *C. albicans* in RPMI 1640 medium was added to the wells of a 96-well polystyrene microplate and incubated at 37 °C for 4 h (early phase) or 24 h (mature biofilm). After that, biofilms were washed three times with PBS to remove non-adherent cells and each well received fresh medium with different concentrations of ToAP2 (100 to 3.12 μM for early phase and 200 to 6.25 μM for mature biofilms); NDBP-5.7 (200 to 6.25 μM for early phase and 400 to 12.5 μM for mature biofilms), amphotericin B (1.08 to 0.03 μM for both early-phase and mature biofilms) or fluconazole (104 to 3.25 μM for both early-phase and mature biofilms) and the plates were incubated at 37 °C for 24 h. Cell viability was evaluated using the alamarBlue reagent, in a concentration of 9%, and fluorescence was detected on a SpectraMax M plate reader (excitation at 550 nm and emission at 585 nm). Cell viability was also analyzed using the fluorescence dye Phloxine B (0.01%) and observing the cells in an inverted microscope (Axio Observer Z1 Carl Zeiss Microscopy, USA) (excitation: 546/12 nm; emission: 607/80 nm). All assays were done in biological triplicates.

### Evaluation of cell membrane integrity of biofilm cells

Membrane permeabilization of *C. albicans* biofilm cells was evaluated using an adaptation of the rhodamine 6 G efflux assay as described by Szczepaniak^42^ One hundred microliters of a cell suspension of *C. albicans* SC 5314 containing 1 × 10^6^ cells/mL was incubated at 37 °C in 96-well microplates for 4 or 24 h, in RPMI medium supplemented with MOPS. After the incubation times, the biofilms were washed three times with PBS and incubated for 2 h in PBS. Afterwards, all the wells received a solution of Rhodamine 6 G in PBS (10 µM) and were incubated for an additional 30 minutes. After this incubation, the biofilms were washed three times with PBS to remove the non-internalized dye, received different concentrations of ToAP2 or NDBP-5.7 diluted in PBS and were incubated for an additional 24 h. Untreated biofilms and biofilms treated with Amphotericin B (1.08 µM in PBS) and Fluconazole (52 µM in PBS) were used as controls. After 24 h, the supernatant was removed, and fluorescence measured in a fluorescence plate reader with excitation at 550 nm and emission at 585 nm (SpectraMax M plate reader). The test was carried out in biological triplicates.

### *Galleria mellonella* infection

*In vivo* toxicity and efficacy against *C. albicans* infection of ToAP2 were tested using *G. mellonella* larvae weighting 250–300 mg as described before^99^. For the toxicity assay, groups of 16 randomly selected larvae were inoculated with different doses of ToAP2 (0, 40, 20 and 10 mg/kg) diluted in PBS in the last proleg using a Hamilton syringe and the larvae were incubated at 37 °C. Larva survival was evaluated daily for 7 days, and larva death was defined by the absence of movement in response to touch^100^. For protection assays, each larva received an inoculum of 5 × 10^5^ *C. albicans* cells and was incubated for 1 h at 37 °C. After that, different doses of ToAP2 peptides diluted in PBS were injected into the infected larvae and the animals were incubated at 37 °C for 7 days. Amphotericin B (2 mg/kg) and PBS alone were used as controls. The assay was performed twice.

### Histopathological analysis of *G. mellonella*

Histopathological evaluation was performed according to a standard protocol^85^. Three larvae from each group (non-infected, infected, infected + ToAP2 40 mg/Kg and infected + Amphotericin B 2 mg/Kg) were used after 24 h of incubation at 37 °C. The larvae were fixed with 10% buffered formalin for 24 h and then transverse and longitudinal incisions were made. The samples were dehydrated with increasing concentrations of ethanol, followed by xylol and incorporated with paraffin. Tissue sections (6 micron) were made with a microtome and placed on glass slides with 5% albumin. The slides were then stained with periodic acid Schiff (PAS) solution and observed in inverted microscope (Axio Observer Z1 Carl Zeiss Microscopy, USA).

### Statistical analyzes

Except for the *G. mellonella* infection assay, which was performed as two biological replicates in different days with similar results, all other analyses were done in biological triplicates performed on different days. ANOVA and Tukey tests were used as post-tests, being considered statistically significant results when p ≤ 0.05. Long-rank was used in the analysis of Mantel-Cox survival curves.

## Supplementary information

Supplementary Information.

Supplementary Information2.

Supplementary Information3.

Supplementary Information4.
