# Supplementary material for: Mechanisms of action of antimicrobial peptides ToAP2 and NDBP-5.7 against Candida albicans planktonic and biofilm cells
Source: Sci Rep. 2020 Jun 25;10:10327. doi: 10.1038/s41598-020-67041-2 (PMC7316759; doi:10.1038/s41598-020-67041-2)
Supplement: Supplementary file 1 — Supplementary Information. [file 41598_2020_67041_MOESM1_ESM.pdf]

Supplementary Figures 1 and 2, and the movie S1 legend

Mechanisms of action of antimicrobial peptides ToAP2 and NDBP-5.7 against *Candida albicans* planktonic and biofilm cells.

Jhones do Nascimento Dias, Calliandra Souza-Silva, Alyne Rodrigues de Araújo, Jessica Maria Teles Souza, Paulo Henrique de Holanda Veloso Júnior; Wanessa Felix Cabral; Maria da Glória da Silva; Peter Eaton, José Roberto de Souza de Almeida Leite, André Moraes Nicola, Patrícia Albuquerque*, Ildinete Silva-Pereira*

* These authors share senior authorship

***Correspondence:**

Ildinete Silva-Pereira
ildinetesp@gmail.com; xocolau@unb.br

Patrícia Albuquerque
palbuquerque@unb.br

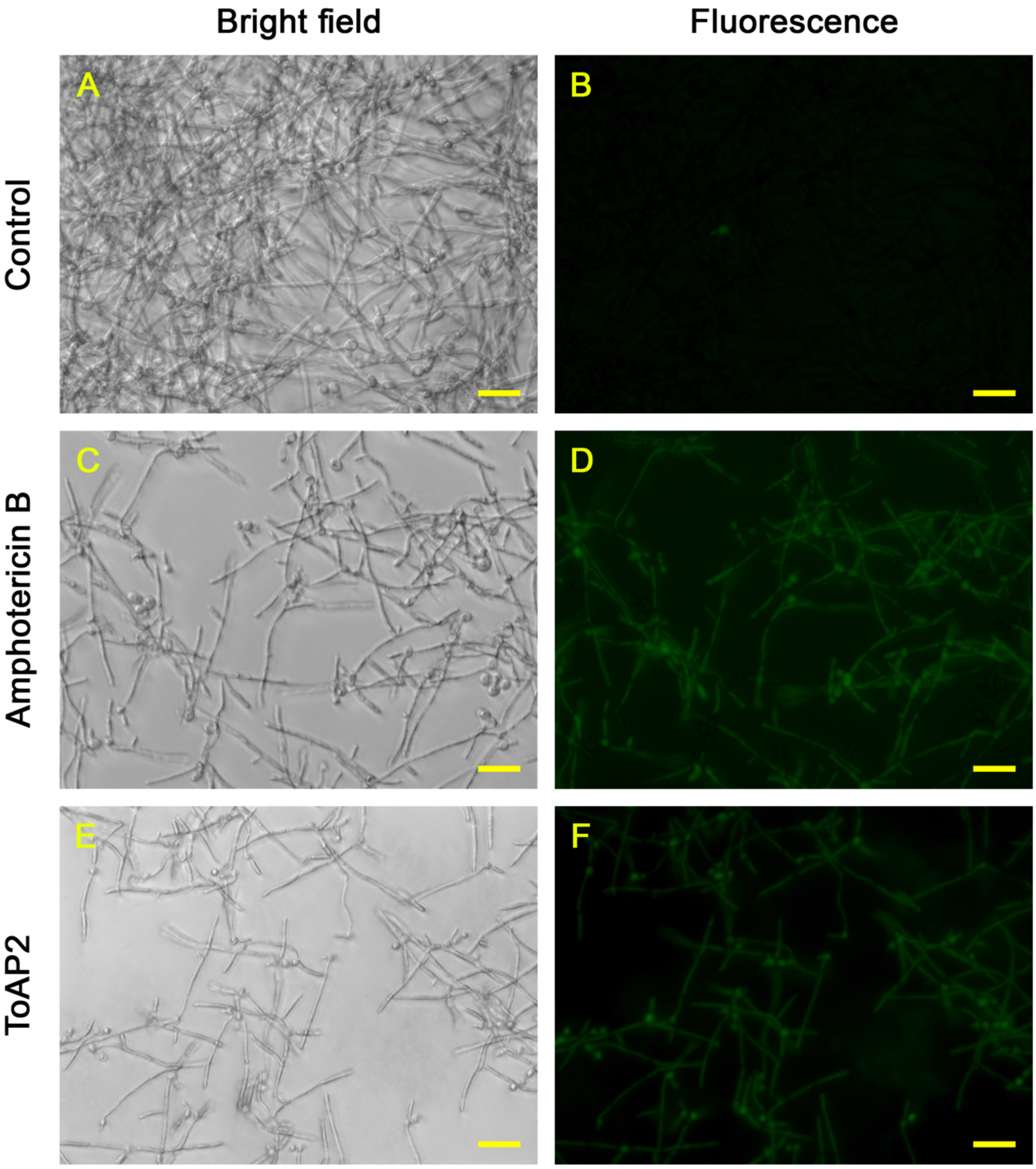

Supplementary Figure 1. Brightfield (A, C and E) and fluorescence (B, D and F) microscopy of *C. albicans* SC 5314 early phase biofilms left untreated (A and B) or treated with 0.5 µM amphotericin B (C and D) or 100 µM ToAP2 (E and F) for 24h. Cell viability was evaluated using the fluorescence dye Phloxine B (0.01%) in an inverted fluorescence microscope (excitation: 546/12 nm; emission: 607/80 nm). Scale bars: 20 µm

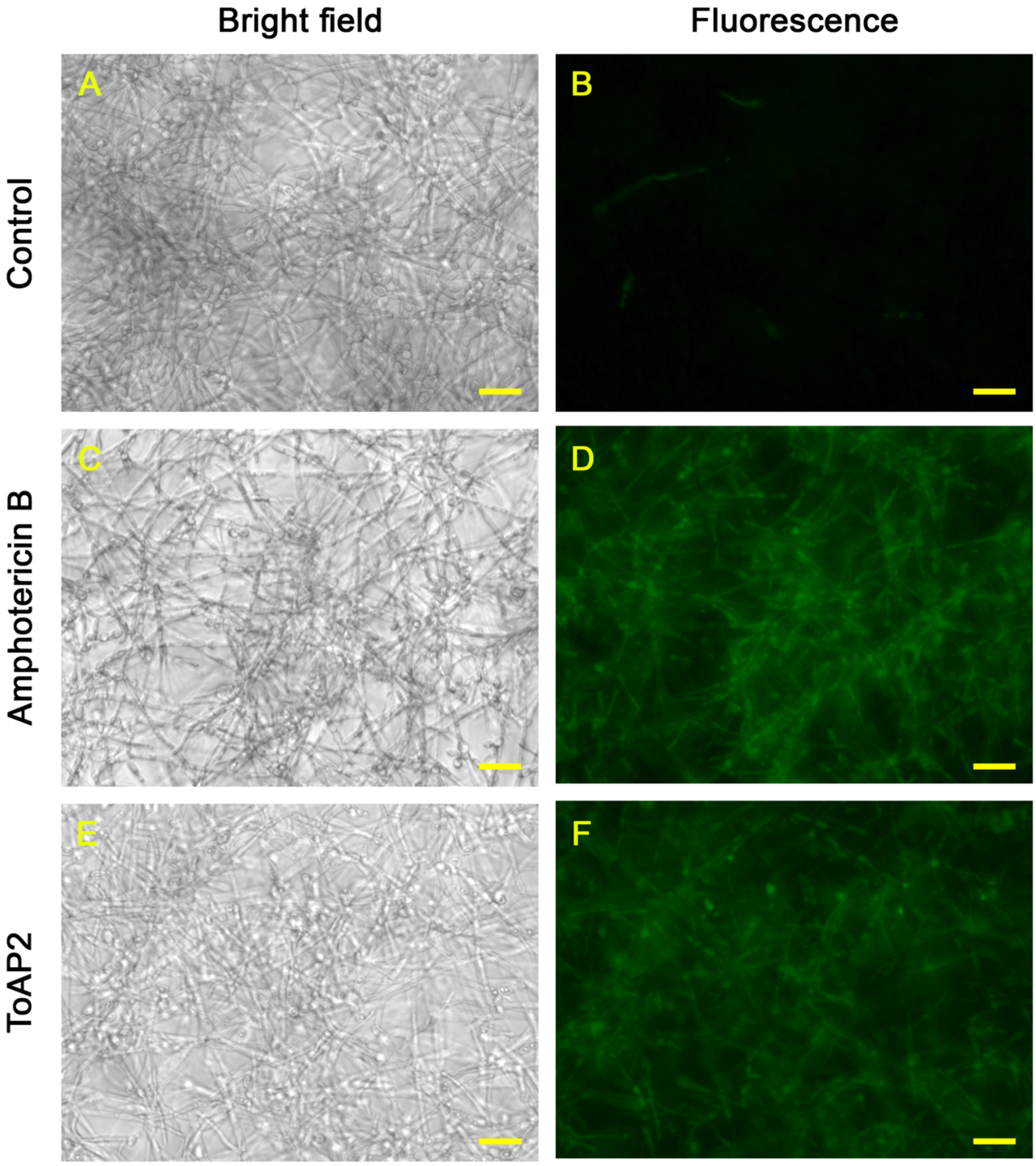

Supplementary Figure 2. Brightfield (A, C and E) and fluorescence (B, D and F) microscopy of *C. albicans* SC 5314 mature biofilms left untreated (A e B) or treated with 2 μM amphotericin B (C e D) or 200 μM ToAP2 (E and F) for 24 h. Cell viability was evaluated using the fluorescence dye Phloxine B (0.01%) in an inverted fluorescence microscope (excitation: 546/12 nm; emission: 607/80 nm). Scale bars: 20 μm

Supplementary Movie S1. Time-lapse microscopy of *C. albicans* SC 5314 filamentation in the presence of MIC and subMIC concentrations of ToAP2 (12.5 μM and 6.25 μM) or NDBP-5.7 (50 μM and 25 μM) during 24 h of incubation in RPMI 1640 medium supplemented with L-glutamine and buffered to pH 7.0 with 165 mM MOPS at 37°C. Bright-field images were taken every 5 minutes and the video was rendered in *Blender 3D* software at 15 fps.
