# Supplementary figures and images for: Mechanisms of action of antimicrobial peptides ToAP2 and NDBP-5.7 against Candida albicans planktonic and biofilm cells
Source: Sci Rep. 2020 Jun 25;10:10327. doi: 10.1038/s41598-020-67041-2 (PMC7316759; doi:10.1038/s41598-020-67041-2)

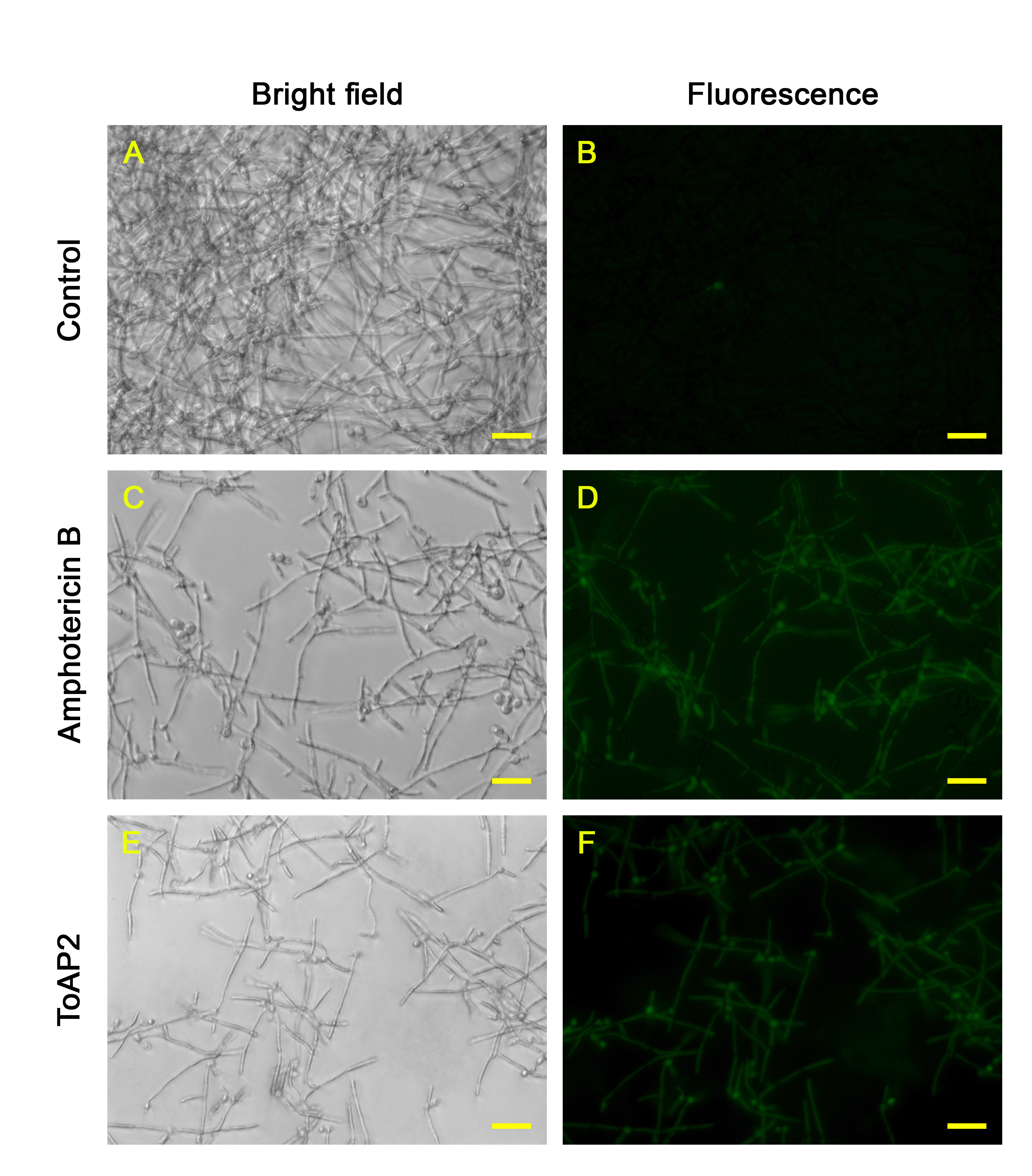

Supplement: Supplementary file 2 — Supplementary Information2. [file 41598_2020_67041_MOESM2_ESM.tif]

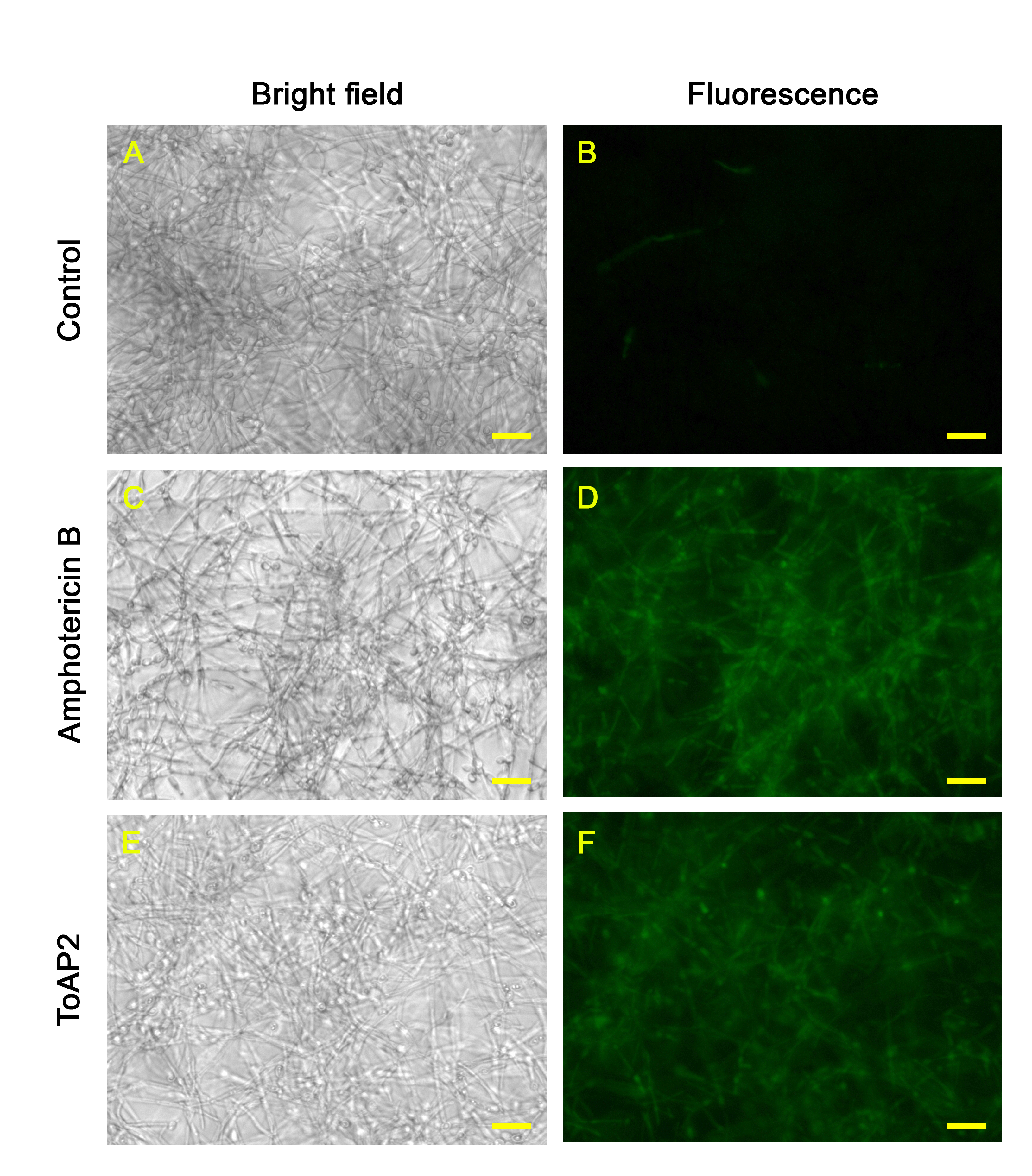

Supplement: Supplementary file 3 — Supplementary Information3. [file 41598_2020_67041_MOESM3_ESM.tif]
